# Effects of single and combined water, sanitation and hygiene (WASH) interventions on nutritional status of children: a systematic review and meta-analysis

**DOI:** 10.1186/s13052-019-0666-2

**Published:** 2019-07-04

**Authors:** Zemichael Gizaw, Alemayehu Worku

**Affiliations:** 1 Department of Environmental and Occupational Health and Safety, Institute of Public Health, College of Medicine and Health Sciences, University of Gondar, Gondar, Ethiopia; 2 Department of Preventive Medicine, School of Public Health, College of Health Sciences, Addis Ababa University, Addis Ababa, Ethiopia

**Keywords:** WASH interventions, Nutritional status of children, Mean height-for-age-z score, Under five children, And developing countries

## Abstract

**Background:**

Under nutrition is linked with poor water, sanitation and hygiene (WASH) condition. However, there is conflicting evidence on the effect of WASH on nutritional status of children. This review was, therefore, conducted to estimate the pooled effect of WASH interventions on child under nutrition.

**Methods:**

All published and unpublished cluster-randomized, non-randomized controlled trials, and before and after intervention studies conducted in developing countries were included. Relevant articles were searched from MEDLINE/PubMed, Cochrane Collaboration’s database, Web of Science, WHO Global Health Library, Google Scholar, Worldcat and ProQuest electronic databases. The methodological quality of the included studies was assessed using JBI critical appraisal checklist for randomized and non-randomized controlled trials. The risk of bias was assessed using the Cochrane Collaboration’s tool for assessing risk of bias in randomized trials. The treatment effect was expressed as standardized mean differences (SMD) with 95% confidence interval (CI).

**Results:**

This meta-analysis of 10 studies including 16,473 children (7776 in the intervention and 8687 in the control group) indicated that WASH interventions significantly associated with increased pooled mean height-for-age-z-score (SMD = 0.14, 95% CI = (0.09, 0.19); I^2^ = 39.3%]. The effect of WASH on HAZ was heterogeneous in age and types of interventions. WASH intervention had more effect on HAZ among under two children [SMD = 0.20, 95% CI = (0.11, 0.29); I^2^ = 37%]. Children who received combined WASH interventions grew better compared with children who received single interventions [SMD = 0.15, 95% CI = (0.09, 0.20); I^2^ = 43.8%].

**Conclusion:**

WASH interventions were significantly associated with increased mean height-for-age-z score in under 5 years old children. The effect of WASH on linear growth is markedly different with age and types of interventions, either single or combined. Implementing combined WASH interventions has a paramount benefit to improve nutritional status of children.

**Electronic supplementary material:**

The online version of this article (10.1186/s13052-019-0666-2) contains supplementary material, which is available to authorized users.

## Background

Children are the most vulnerable group to a wide range of infections unless special attention is given. The highest proportions of infections among children are poor WASH related diarrheal and parasitic diseases. Globally, there are nearly 1.7 billion cases of childhood diarrheal disease every year and diarrhea is responsible for killing around 525,000 children every year [1]. About 3.5 billion people (the majority of these cases were children) in the world were infected with intestinal parasites caused by helminthes and protozoa during 2009 [2].

Repeated exposure to diarrheal and parasitic infections causes environmental enteropathy (EE) or sometimes called environmental enteric dysfunction (EED). EE is an inflammatory condition of the gut of children which is characterized by villous atrophy, crypt hyperplasia, increased permeability, inflammatory cell infiltrate, and modest malabsorption [3–5]**.**

Diarrhea, parasitic infections and EE are key mediating pathways linking poor WASH to developmental deficit [6–8]**.** A large body of evidence suggests that malnutrition is linked with poor WASH practice [9–12]. Poor WASH is associated with under nutrition as a result of diarrhea, nematode infection and EE. Diarrhea and intestinal worms cause nutrient losses and diversion of nutrients from growth to the immune system to fight the infection [13–19]. EE increases the small intestine’s permeability and reduces nutrient absorption [20–24]. The link of poor WASH and under nutrition has also socio-economical mechanism. For instance the energy cost of carrying water for long distances from the source to home. The average woman carrying a typical load of 20 l on level ground would consume about 39 cal per kg of body weight per hour with an assumption that 1 g of maize meal yields 3.5 cal [25]**.**

WASH interventions are the most holistic and effective approaches to prevent stunting and wasting among under two children. However, there is conflicting evidence on the effect of WASH on linear growth. Some studies reported that there is no significant association between WASH and linear growth [26–30] and some others reported that WASH has significant effect [8, 31–33]. This systematic review and meta-analysis was, therefore, aimed to estimate the pooled effect of WASH on linear growth among under five children.

## Methods

### Research question

Does access to improved WASH facilities have effect on child growth?

### Criteria for considering studies for this review

#### Types of studies

All published and unpublished community-based trials (including cluster-randomized, quasi-randomized, non-randomized controlled trials, controlled before and after intervention studies) conducted in developing countries to analyze the effect of WASH on the mean height-for-age- z score were included. Studies published in English language in the last 10 years were also included. Citations with no abstracts and/or full texts, duplicate studies, and studies with poor quality were excluded.

#### Types of participants

Children aged under 5 years.

#### Types of interventions

1. Any intervention aimed at improving the microbiological quality of drinking water, including household and community level water treatment, water source protection and household water handling.
2. Interventions aimed to reduce direct and indirect contact with human faeces (pour flush, water sealed flush toilet, piped sewer system, septic tank, simple pit latrines, VIP latrine or use off scoop for the disposal of child faeces).
3. Interventions aimed at the promotion of hand washing with soap or ash after defecation, disposal of child faeces and prior to preparing and handling food
4. Any WASH promotion aimed at WASH behavioral change like community-led total sanitation (CLTS)
5. Any combination of the WASH interventions listed above

#### Control

1. Water quality: participants who continued with usual practice, or a less stringent version of the intervention (i.e. new protected well but no household disinfection).
2. Sanitation: participants who continued with usual practice rather than following the prescribed intervention.
3. Hygiene: no hand washing promotion and participants who continued with usual practice.
4. WASH promotion: participants who did not receive WASH behavioral change education

#### Types of outcome measures

Child nutritional status or stunting measured by mean height-for-age-z score.

### Search strategy and study selection

We searched relevant articles from MEDLINE/PubMed, Cochrane Collaboration’s database, Web of Science, WHO Global Health Library, Google Scholar, Worldcat and ProQuest. A three-step search strategy was utilized in this review. An initial limited search of MEDLINE was undertaken followed by analysis of the text words contained in the title and abstract, and of the index terms used to describe articles. A second search using all identified keywords and index terms was undertaken across all included databases. Thirdly, references of all identified articles were searched to get additional studies. The below box shows terms used to search literatures.

**Table:** 

(((“child”[MeSH Terms] OR “child”[All Fields] OR “children”[All Fields]) OR (“child”[MeSH Terms] OR “child”[All Fields])) AND ((((((((“sanitation”[MeSH Terms] OR “sanitation”[All Fields]) OR “Wastemanagement”[All Fields]) OR “Latrine utilization”[All Fields]) OR “Water quality”[All Fields]) OR “Food hygiene”[All Fields]) OR “Personal hygiene”[All Fields]) OR “Hand washing”[All Fields]) OR “Water, sanitation and hygiene”[All Fields])) AND ((((((((“malnutrition”[MeSH Terms] OR “malnutrition”[All Fields] OR “undernutrition”[All Fields]) OR (“malnutrition”[MeSH Terms] OR “malnutrition”[All Fields])) OR (“growth disorders”[MeSH Terms] OR (“growth”[All Fields] AND “disorders”[All Fields]) OR “growth disorders”[All Fields] OR “stunting”[All Fields])) OR “Linear growth deficit”[All Fields]) OR “Growth faltering”[All Fields]) OR “Growth failure”[All Fields]) OR “Growth impairment”[All Fields]) OR “Growth disorder”[All Fields])	

### Assessment of methodological quality

Search results from different electronic databases were exported to Endnote reference manager to remove duplication. We screened out articles using titles and abstracts. We further investigated and assessed full-text articles against the inclusion and exclusion criteria. The methodological quality of the included studies was assessed using JBI critical appraisal checklist for randomized and non-randomized controlled trials (Additional file 1) [34].

### Data extraction

We independently extracted data from papers included in the review using the JBI standardized data extraction tool (Additional file 2). The data extraction form was piloted on randomly selected papers and modified accordingly. Eligibility assessment was performed independently by the two reviewers. We invested our maximum effort to avoid introduction of errors (e.g. entering wrong numerals into a spreadsheet and failure to identify required data from a study report) or bias during extraction. Data were systematically extracted relating to the nature of studies.

### Assessment of risk of bias in included studies

We independently assessed the risk of bias of included studies using the Cochrane Collaboration’s tool for assessing risk of bias in randomized trials [35]. Random sequence generation (selection bias), allocation concealment (selection bias), blinding of participants and personnel (performance bias), blinding of outcome assessment (detection bias), incomplete outcome data (attrition bias), selective reporting (reporting bias) and other possible risk of bias (buffer to prevent information contamination and measure or adjust potential confounders) were items used to assess risk of bias. We assessed studies for each item with answers of ‘low’ indicating low risk of bias, ‘high’ indicating high risk of bias and ‘unclear’ indicating either lack of information or uncertainty over the potential for bias (Additional file 3).

### Measures of treatment effect

Stata version 11 was used to measure treatment effect and to conduct other analysis. We expressed treatment effect sizes as SMD with 95% CI using their original scale. We used forest plot to present results.

### Sensitivity analysis

Sensitivity analysis was carried out to see the impact of individual studies for the pooled result.

### Assessment of heterogeneity

We assessed statistical heterogeneity using the I^2^ statistics. Galbraith plot was also used to observe heterogeneity.

### Assessment of publication bias

Funnel plot was used to see publication bias subjectively and Egger’s test was used to objectively check potential publication bias.

### Dealing with missing data

We contacted authors of potentially relevant studies that did not report the outcome and asked them to provide information on the availability of outcome data. Articles with incomplete data were excluded from the meta-analysis while they were included in the systematic review.

## Results

### The search process

**Fig. 1:**
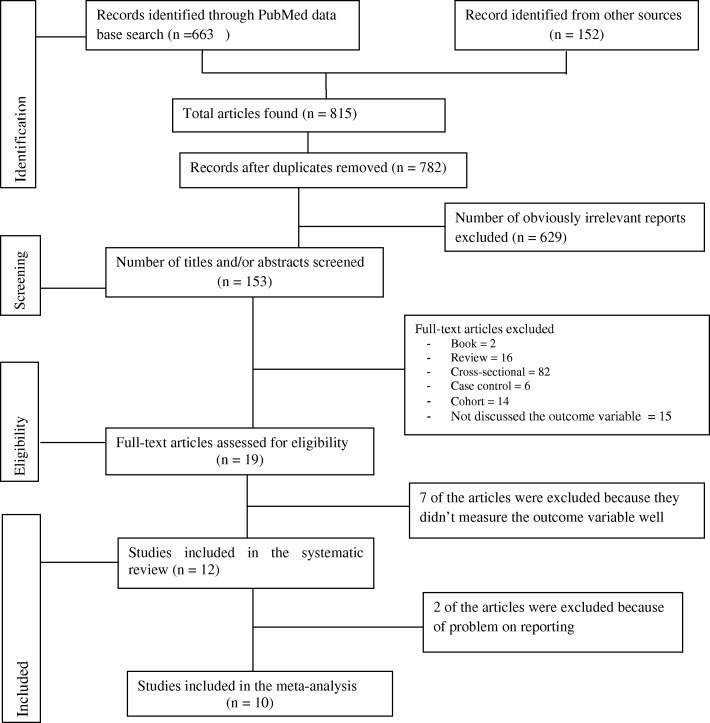
Study selection flow diagram

The search strategy identified 815 titles and abstracts (663 from PubMed and 152 from other sources) published before 11 August 2018. We obtained 782 articles after we removed duplicated articles. Following assessment by title and abstract, 153 articles were retrieved for more evaluation. Twelve articles were included for systematic review and 10 articles were included for meta-analysis based on the inclusion criteria. Of the 12 included reports, 9 were published in journals, one was a CEDLAS report, one was a Feinstein International Center report and one was dissertation. All of the included studies were published in English. The study selection process is outlined in Fig. 1.

### Description and characteristics of included studies

Two studies [Arnold, 2009 and Langford, 2011] were non-randomized trial and the rest were randomized trial. All included studies were conducted in low-income or middle income country settings. Two studies were conducted in Mali [Alzua, 2015 and Pickering, 2015], two in Uganda [Marshak, 2015 and Muhoozi, 2017], two in India [Clasen, 2014 and Patil, 2014], One in Bangladesh [Shafique, 2013]*,* one in Kenya [Arnold, 2018], one in Pakistan [Bowen, 2012]*,* one in Guatemala [Arnold, 2009], one in Cambodia [McGuigan, 2011] and one in Nepal [Langford, 2011].

Intervention duration was ranged from 6 to 36 months. Three interventions were implemented for 6 months [Muhoozi, 2017; Marshak, 2015 and Langford, 2011], One intervention for 9 months [Bowen, 2012], one intervention for 10 months [Alzua, 2015], two interventions for 12 months [Shafique, 2013 and McGuigan, 201], one intervention for 13 months [Clasen, 2014], one intervention for 17 months [Pickering, 2015], one intervention for 21 months [Patil, 2014], one intervention for 24 months [Arnold, 2018], and one intervention for 36 months [Arnold, 2009]. Two studies [Alzua, 2015 and Pickering, 2015] implemented CLTS, one study [Patil, 2014] implemented total sanitation campaign (TSC), one study [Arnold, 2018] implemented WASH and nutrition, two studies [Marshak, 2015 and Muhoozi, 2017] implemented nutrition and food hygiene, one study [Arnold, 2009] implemented water treatment and hand washing, one study [Shafique, 2013] implemented nutrition, health and hygiene education and hand hygiene, two studies [Bowen, 2012 and Langford, 2011] implemented hand washing, one study [McGuigan, 2011] implemented home based water treatment, and one study [Clasen, 2014] implemented latrine construction and promotion.

Height-for-age-z score was reported in all studies. Except two studies [McGuigan, 2011 and Arnold, 2018]*,* all others measured weight-for-age z-scores, five studies [Alzua, 2015; Arnold, 2018; Marshak, 2015; Pickering, 2015 and Shafique, 2013] reported stunting, three studies [Alzua, 2015; Marshak, 2015 and Pickering, 2015] reported underweight, three studies [Muhoozi, 2017; Patil, 2014 and Arnold, 2009] reported mid upper arm circumference z-scores, one study [Muhoozi, 2017] reported head circumference z-scores, two studies [Bowen, 2012 and Patil, 2014] reported body mass index, nine studies [Alzua, 2015; Arnold, 2018; Bowen, 2012; Clasen, 2014; Pickering, 2015; Arnold, 2009; Langford, 2011; McGuigan, 2011; and Shafique, 2013] reported diarrhea, five studies [Alzua, 2015; Pickering, 2015; Arnold, 2009; Langford, 2011 and Shafique, 2013] reported respiratory illness, two studies [Clasen, 2014 and Patil, 2014] reported STHs, one study [Patil, 2014] reported anemia, three studies [Arnold, 2018; Bowen, 2012 and Muhoozi, 2017] reported child development quotients, and one study [Arnold, 2018] reported markers of EE.

### Risk of bias in included studies

#### Allocation

With the exclusion of the two non-randomized studies [Arnold, 2009 and Langford, 2011], we judged sequence generation is adequate in all other studies. All other studies allocated the intervention and control groups randomly. Random number generator [Arnold, 2018; Bowen, 2012 and Pickering, 2015], computer generated sequence [Clasen, 2014 and Shafique, 2013] and raffle system [McGuigan, 201]) were used to generate random sequence. However, allocation concealment was not possible in all studies except Bowen, 2012.

#### Blinding

Four of the include studies [Alzua, 2015; Bowen, 2012; Clasen, 2014 and Muhoozi, 2017] made study participants or personnel blind and we judged these studies to be at low risk of performance bias. Alzua, 2015; Arnold, 2018; Bowen, 2012; Clasen, 2014 and Pickering, 2015 concealed group allocation from personnel who measured outcome variables in and thus judged to be at low risk of detection bias.

#### Incomplete outcome data

There was lose of follow-up in all studies, however the majority of studies [Alzua, 2015; Arnold, 2018; Clasen, 2014; McGuigan, 2011; Muhoozi, 2017; Patil, 2014; Pickering, 2015 and Shafique, 2013] balanced participants in both arms and conducted intention-to-treat analysis to manage attrition bias and we judged these studies to be at low risk of attrition bias.

#### Selective reporting

We assessed the trial registries and protocols of six studies; Arnold, 2018 and McGuigan, 2011 were not reported as per protocol. We found Alzua, 2015; Bowen, 2012; Patil, 2014 and Shafique, 2013 reported as per protocol.

#### Other potential sources of bias

**Fig. 2:**
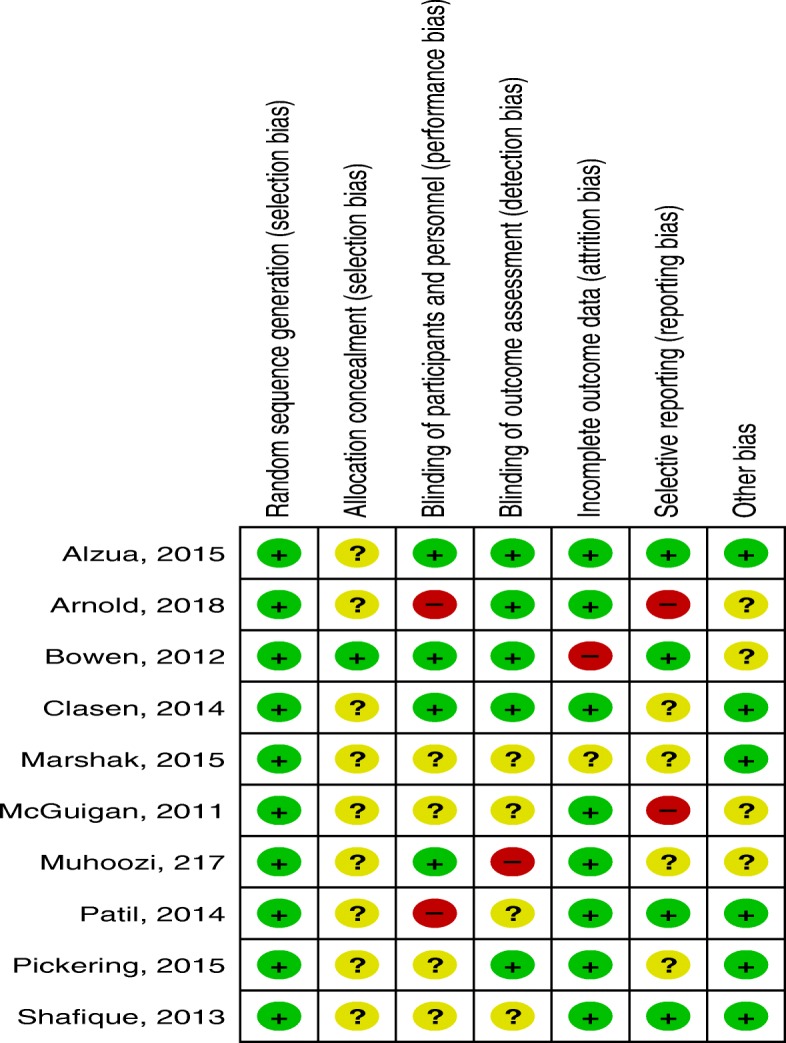
Risk of bias summary: review authors’ judgments about each risk of bias item for each included study

**Fig. 3:**
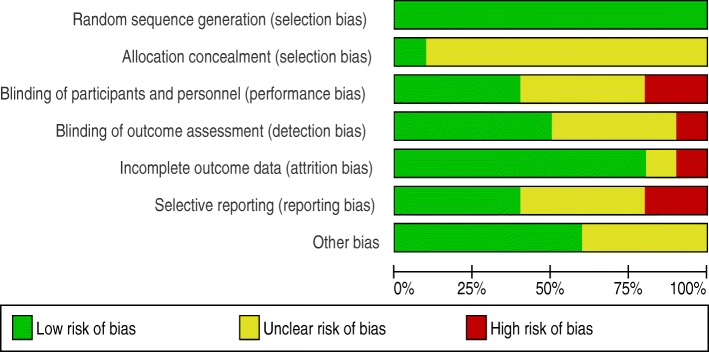
Risk of bias graph: review authors judgment about each risk of bias item presented as percentages across all included studies

We assessed authors’ investment to prevent information cross contamination among clusters and potential effect of confounders. Alzua, 2015; Marshak, 2015 and Pickering, 2015 used buffer to prevent information contamination and Patil, 2014 and Shafique, 2013 measured and adjusted anticipated confounders (see Figs. 2 and 3 for the risk summary of studies).

#### Effects of WASH interventions on linear growth

Different community-based randomized and non-randomized trials discussed the effect of single or/and combined WASH interventions on child linear growth (expressed as mean height-for-age-z score in this study). The effect of WASH interventions on mean height-for-age-z score is summarized below.

Alzua, 2015 was a cluster-randomized controlled trial conducted among 7328 (3564 in the control arm and 3764 in the intervention arm) children in Mali. This study reported that children in CLTS villages were taller (0·18 increase in height-for-age- z score, 95% CI 0.03–0.32) and less likely to be stunted (PR 0·86, 95% CI 0·74–1·0) compared to children in the control arm.

Arnold, 2009 was a non-randomized intervention conducted among 877 children under 5 years of age in Guatemala. Compared to children in the control, a water quality and hand washing intervention had no effect on weight- for-age- z score (MD = − 0.053, 95% CI = (− 0.331, 0.206), weight-for-height-z score (MD - 0.066, 95% CI = (− 0.248, 0.124), height-for-age- z score (MD = 0.041, 95% CI = (− 0.305, 0.326) or mid-upper arm circumference (cm) (MD = − 0.014, 95% CI = (− 0.166, 0.145).

Arnold, 2018 was a cluster-randomized controlled trial conducted in Kenya among 2101children (364 in the intervention arm and 1737 in the control arm) and in Bangladesh among 712 children (199 in the intervention arm and 513 in the control arm). The study reported that children who had access to improved latrine were taller [MD = 0·15, 95% CI = (0·02–0·28] in Kenya and [MD = 0·22, 95% CI = (0·03–0·40)] in Bangladesh.

Bowen, 2012 was a cluster-randomized controlled trial conducted in Pakistan among 461 children (160 in the control group, 141 in the hand washing group and 160 in hand washing and water treatment group). This study found that 24.9% (95% CI, 20.0–30.6%) and of children had z scores that were more than 2 SDs below the expected z score for height for age which did not differ significantly across study groups.

Clasen, 2014 was a cluster-randomized controlled trial conducted in India among 24, 969 individuals in intervention villages and 25, 982 individuals in control villages. The study reported that the mean height-for-age-z-score was not significantly differ between children from households with functional latrine and with no functional latrine [MD = − 0·06, 95% CI = (− 0·27 to 0·15)].

Langford, 2011 was a non-randomized controlled trial study among 88 children under 1 year of age in Nepal to see the effect of hand washing on subclinical infections and growth. The study reported that hand washing intervention had no effect on weight-for-age z-score (MD = − 0.24, 95% CI = (− 0.76, 0.28), weight-for-height z-score (MD = − 0.11, 95% CI = (− 0.53, 0.31) or height-for-age z-score (MD = − 0.13, 95% CI = (− 0.54, 0.28).

Marshak, 2015 was a randomized control trial conducted in Uganda to see the impacts of a program entitled “Community Resilience to Acute Malnutrition”. This study included 1762 children in the control group (647 children in the baseline, 572 children in the midline, 543 in the endline) and 1656 children in the WASH intervention group (614 children in the baseline, 555 children in the midline, and 487 in the endline). The report of the assessment indicated that children living in intervention settlements were less likely to be stunted compared to children living in non-intervention settlements. This relationship is further supported by a significant difference between intervention (HAZ = − 1.07) and non-intervention (HAZ = − 1.27) settlements.

McGuigan, 2011 was a cluster-randomized controlled trial conducted among 928 children (nutrition outcome data were available for 760 children) under 5 years of age in Cambodia to the impact of solar disinfection of drinking water on childhood diarrhea. Compared to children in the control arm, a water quality (SODIS) intervention had no effect on weight-for-age- z score (MD = 0.26, 95% CI = (− 0.01, 0.53), weight-for-height- z score (MD = 0.15, 95% CI = (− 0.15, 0.45) or height-for-age- z score (MD = 0.22, 95% CI = (− 0.04, 0.48).

Muhoozi, 2017 was a community-based, open cluster-randomized trial conducted in Uganda among 511 children aged 6–8 months (263 in the intervention group and 248 in the control group). This study revealed that there was no evidence of a difference in mean length-for-age- z score at 20–24 months between the two study groups [MD = 0.10, 95% CI = (− 0.17, 0.36)].

Patil, 2014 was a cluster-randomized controlled trial conducted in India among 5209 children (2609 in the control arm and 2600 in the intervention arm) to see the effect of India’s total sanitation campaign on defecation behaviors and child health. The study depicted that the intervention had no significant effect on length/height-for-age-z score (HAZ in the control group = − 1.38 and in the intervention group = − 1.81).

Pickering, 2015 was a cluster-randomized controlled trial conducted in Mali to see the effect of CLTS on child diarrhea and child growth. Researchers enrolled 2365 households to receive the CLTS intervention and 2167 households to the control group. The finding of the study showed that children in CLTS villages were taller (0·18 increase in height-for-age-z score, 95% CI 0·03–0·32) and less likely to be stunted (35% versus 41%, PR 0·86, 95% CI 0·74–1·0) than children in control villages.

Shafique, 2013 is a randomized controlled trial conducted in Bangladesh to measure the relative effect of directed use of benzalkonium chloride-containing, water-based hand sanitizers and broad-range multiple micronutrient powder (MNP) along with nutrition, health and hygiene education (NHHE) to prevent infections and linear growth faltering reported that combined intervention of directed hand-sanitizer use and micronutrient powder along with NHHE significantly improved linear growth of low birth weight infants compared to NHHE alone.

**Fig. 4:**
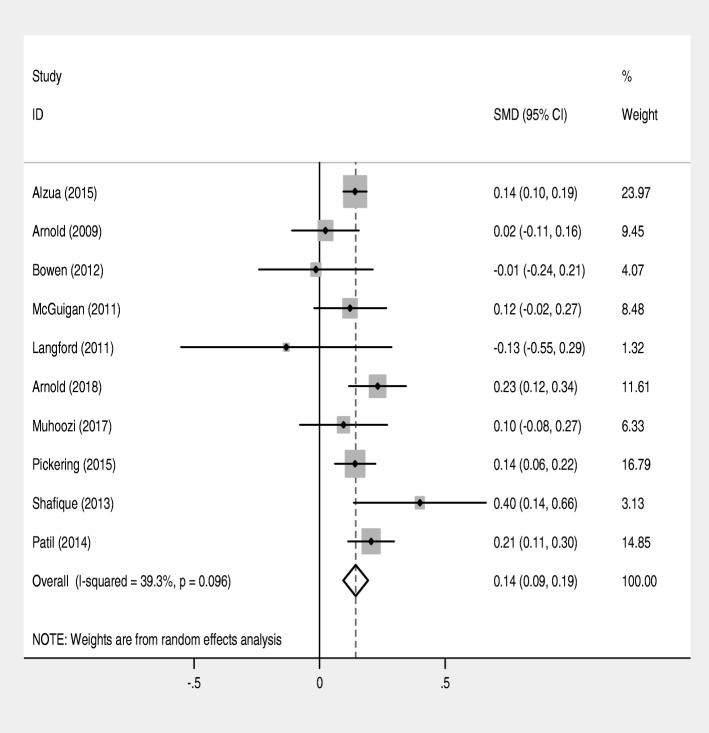
Forest plot of comparison: height-for-age-z score (all community-based trial studies)

This systematic review and meta-analysis of 10 studies including 16,473 children (7776 in the intervention group and 8687 in the control group) indicated that single or/and combined WASH interventions significantly associated with increased mean height-for-age-z-score (SMD = 0.14, 95% CI = (0.09, 0.19); I^2^ = 39.3%] (Fig. 4).

#### Assessment of heterogeneity

**Fig. 5:**
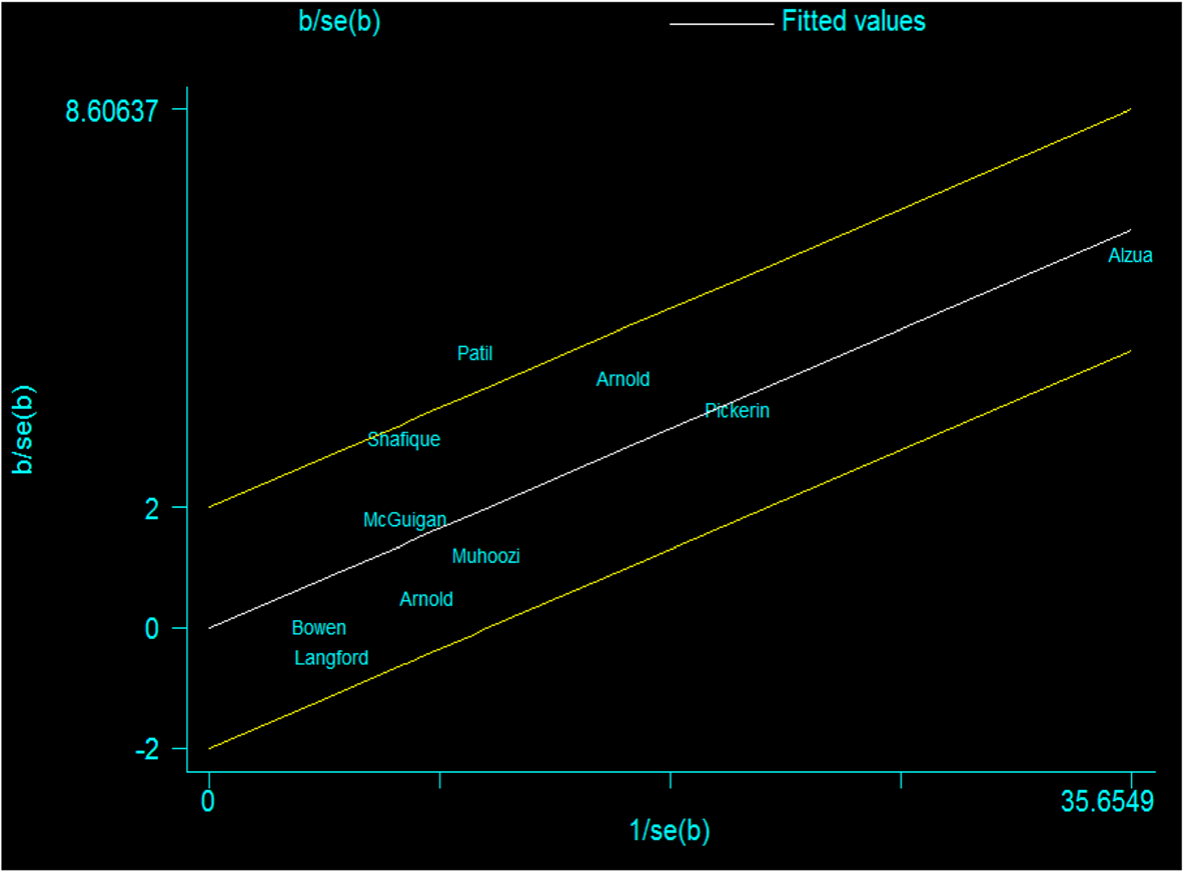
Galbraith plot for heterogeneity

The I^2^ statistics of the aggregate pooled estimate (I^2^ = 39.3%) showed moderate heterogeneity. As a result, Galbraith plot was used to observe heterogeneity. The plot indicated that Patil S, 2014 caused the heterogeneity (Fig. 5).

#### Subgroup analysis

**Fig. 6:**
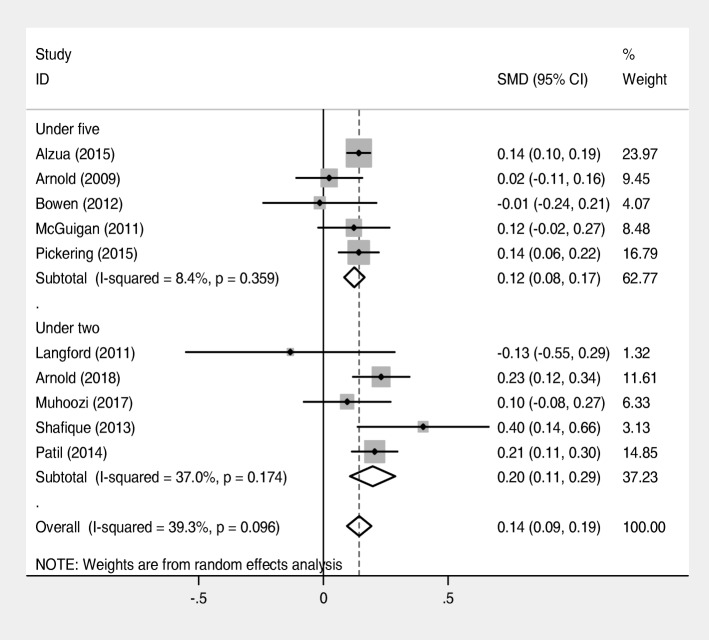
Forest plot of comparison: height-for-age-z score (subgroup analysis by age)

A subgroup analysis by age showed that single or/and combined WASH intervention had more effect on mean height-for-age-z score among under two children compared with under 5 years old children[SMD = 0.20, 95% CI = (0.11, 0.29); I^2^ = 37%] (Fig. 6).

**Fig. 7:**
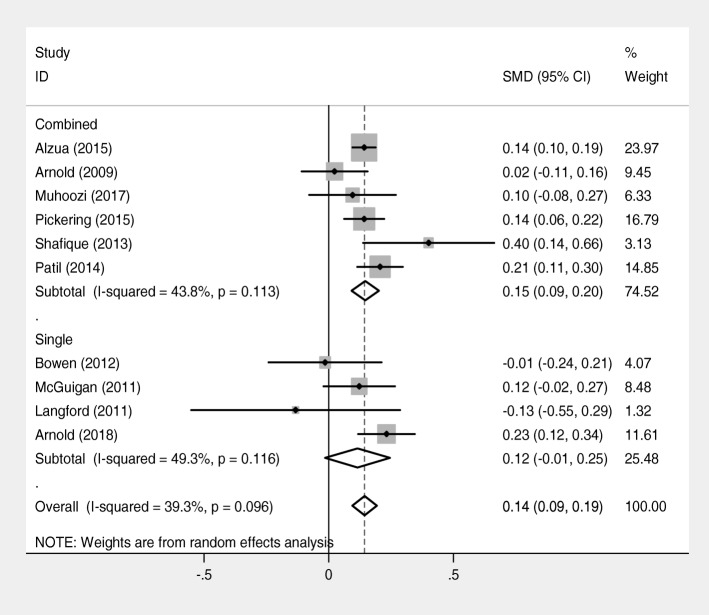
Forest plot of comparison: height-for-age-z score (subgroup analysis by intervention)

A subgroup analysis by type of WASH interventions (single interventions and combined interventions) was done. The result of the analysis showed that combined WASH interventions significantly associated with increased mean height-for-age-z score. Children who received combined WASH interventions grew better compared with children who received single interventions [SMD = 0.15, 95% CI = (0.09, 0.20); I^2^ = 43.8%] (Fig. 7).

#### Sensitivity analysis

**Fig. 8:**
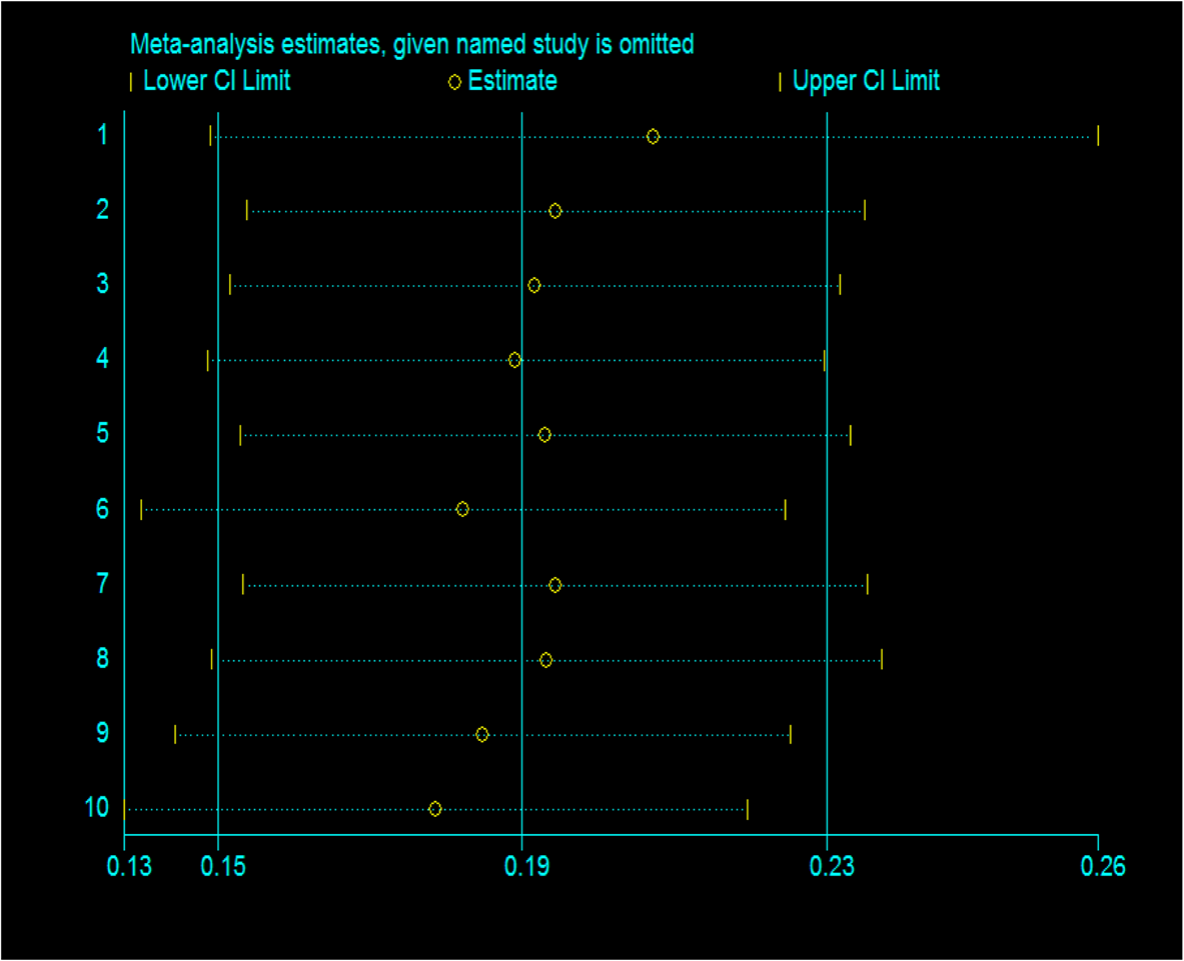
Sensitivity analysis to assess the impact of individual studies to the mean height-for-age-z score pooled estimate

The sensitivity analysis test result showed that the pooled effect of WASH intervention on the mean height-for-age-z score was 0.19 [SMD = 0.19, 95% CI = 0.15, 0.23]. There are studies which become out of the pooled result when they are out of the analysis. The maximum impact for these studies was from 0.13 to 0.26, which is a 95% CI. There was no any significant difference of each study for the total pooled result (Fig. 8).

#### Publication bias

**Fig. 9:**
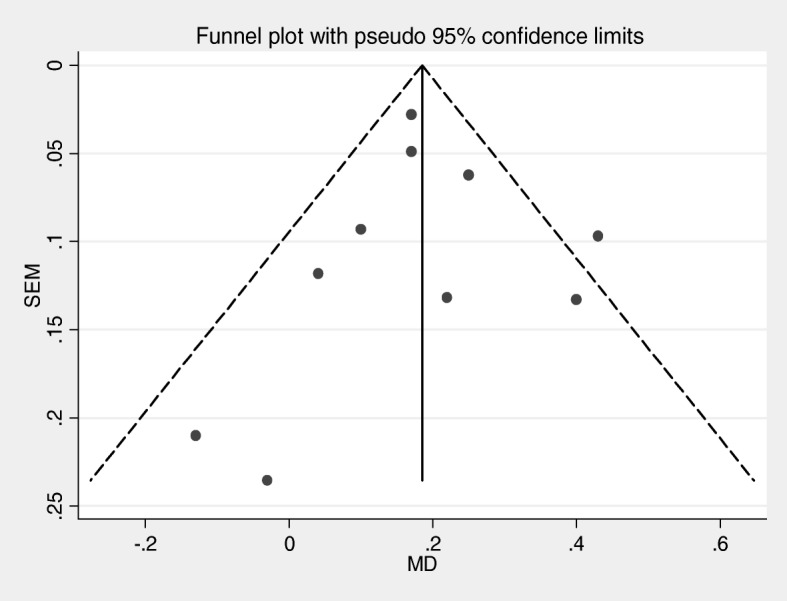
Funnel plot with pseudo 95% CI to show publication bias

The funnel plot (Fig. 9) and Egger’s test (Table 1) show that there was no publication bias. The funnel plot is symmetric and *p* value for Egger’s test is 0.988.

**Table 1:**
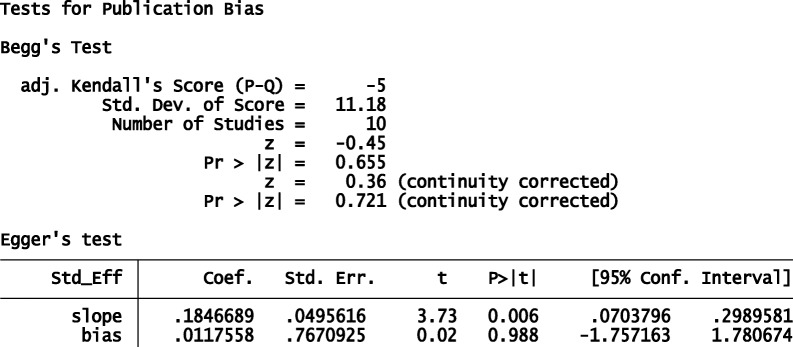
Egger's test showing objectively testing of bias

## Discussion

### Summary of evidence

Twelve studies are included in this review. The review included ten community-based cluster-randomized controlled trials and two non-randomized community based trials. Studies included various water, sanitation and hygiene (WASH) interventions either singly or in combination. Most of the included studies were considered to be at low risk of bias. Height-for-age-z-score, the primary outcome variable of this review was available from all studies. However, two of the studies didn’t report SD for the mean height. As a result, the meta-analysis was limited to data from ten studies.

In this systematic review and meta-analysis, WASH interventions significantly associated with increased height-for-age z-scores in children. The report of this review is consistent with the reports of other systematic reviews. For instance, a systematic review and meta-analysis which included seven RCT comparators reported that WASH intervention was marginally associated with mean height-for-age-z score (MD = 0.08, 95% CI = 0.00–0.16)) [36]. Similarly, a review conducted on the impact of combined WASH intervention on growth, non-diarrheal morbidity and mortality in children residing in low and middle-income countries found that WASH interventions improved height-for-age-z scores (MD = 0.22; 95% CI 0.12, 0.32) and decreased the risk of stunting by 13% (RR = 0.87; 95% CI = 0.81, 0.94)) [37]. Other individual studies also reported that WASH interventions were significantly associated with children’s height [38–41]. The link of WASH and increased mean height-for-age-z score is due to improved WASH condition prevents diarrhea and parasitic infections which could cause reduced absorption and nutrient losses, reduced appetite, and diversion of energy and nutrients from growth to the immune system to fight the infection [13–19]. Improved WASH prevents EE which increases the small intestine’s permeability and reduces nutrient absorption [20–24].

This systematic review and meta-analysis indicated that WASH interventions are more effective to improve childhood nutrition among under two children compared with interventions in under five children. This might be due to the fact that the first 2 years of life are the window to child’s future. Exposed to sanitation related diarrheal and intestinal parasitic infections in the first 1000 days of a child’s life causes irreversible, long-term damage to a child’s health. Nutritional interventions during this time [42, 43].

We found that combined WASH interventions were more effective to improve child nutritional status than single interventions. This can be justified that the exposures to faecal-oral pathogens through drinking water, sanitation or hygiene, which is the mediating pathway to WASH and nutrition is complex. Hand washing or treatment of drinking water or food safety or sanitation alone cannot prevent the occurrence of faecal-oral diseases. The integration of interventions is effective to prevent infections and to promote nutrition status [44, 45].

### Applicability of evidence

There is suggestive evidence from cluster-randomized controlled trials on the effect of WASH interventions on nutritional status of children. However, there is limited evidence on the effect of WASH interventions on nutritional outcomes in children. We believe, the result of this meta-analysis provides supportive evidence on the effect of WASH interventions on nutritional status of children. The finding of this review provides evidence for policy makers, health practitioners, WASH and nutrition advocators to enhance child health, child nutrition and sanitation condition, and to prevent sanitation related infectious diseases. Further large scale studies designed to measure the impact of WASH interventions on nutritional outcomes in children are needed.

### Limitation

We entirely relied on freely access electronic databases to search relevant articles. We didn’t include articles available in hard copy. During the process of the review, we identified one study that collected but did not report nutritional outcomes in children. It is possible that other studies which we did not identify collected data on child nutritional status and WASH that we have been unable to include in this review. Due to these, the searching was not exhaustive. We believed we could get more relevant articles if we had access to other databases and hard prints.

## Conclusion

WASH interventions were significantly associated with increased mean height-for-age-z score in under 5 years old children. The effect of WASH on linear growth is markedly different with age and types of WASH interventions, either single or combined. Implementing combined WASH interventions has a paramount benefit to improve nutritional status of children.

## Additional files

Additional file 1: JBI critical appraisal checklist for randomized controlled trials. (DOCX 31 kb)

Additional file 2: Data extraction format. (DOCX 35 kb)

Additional file 3: Criteria for judging risk of bias in the ‘Risk of bias’ assessment tool. (DOCX 59 kb)

## Data Availability

The extracted data will be made available upon requesting the primary author.

## Abbreviations

CI: Confidence interval
CLTS: Community leading total sanitation
c-RCT: Clustered randomized controlled trial study
HAZ: Height-for-age-z score
HS: Hand sanitizers
JBI: The Joanna Briggs Institute
MD: Mean difference
MNP: Multiple micronutrient powder
NHHE: Nutrition, health and hygiene education
RCT: Rndomized controlled trial
SD: Standard deviation
SMD: Standardized mean difference
TS: Total sanitation
VIP latrine: Ventilated improved latrine
WASH: Water, sanitation and hygiene
WAZ: Weight-for-age-z score
WHO: World health organization
