# Supplementary material for: Effects of single and combined water, sanitation and hygiene (WASH) interventions on nutritional status of children: a systematic review and meta-analysis
Source: Ital J Pediatr. 2019 Jul 4;45:77. doi: 10.1186/s13052-019-0666-2 (PMC6610930; doi:10.1186/s13052-019-0666-2)
Supplement: Supplementary file 1 — JBI critical appraisal checklist for randomized controlled trials. (DOCX 31 kb) [file 13052_2019_666_MOESM1_ESM.docx]

## JBI critical appraisal checklist for randomized controlled trials

| Criteria | Included papers | | | | | | | | | |
| --- | --- | --- | --- | --- | --- | --- | --- | --- | --- | --- |
|  | Alzua , 2015 | Arnold , 2018 | Bowen , 2012 | Clasen , 2014 | Marshak , 2015 | McGuigan, 2011 | , Muhoozi, 2017 | Patil, 2014 | Pickering , 2015 | Shafique, 2013 |
| 1. Was the assignment to treatment groups truly random? | 🗸 | 🗸 | 🗸 | 🗸 | 🞨 | 🞨 | 🗸 | 🗸 | 🗸 | 🗸 |
| 1. Were participants blinded to treatment allocation? | NA | NA | 🗸 | NA | NA | NA | NA | NA | NA | NA |
| 1. Was allocation to treatment groups concealed from the allocator? | 🗸 | 🞨 | 🗸 | 🗸 | ? | ? | 🗸 | 🞨 | ? | ? |
| 1. Were the outcomes of people who withdrew described and included in the analysis? | 🗸 | 🗸 | 🞨 | 🗸 | ? | 🗸 | 🗸 | 🗸 | 🗸 | 🗸 |
| 1. Were those assessing the outcomes blind to the treatment allocation? | 🗸 | 🗸 | 🗸 | 🗸 | ? | ? | 🞨 | ? | 🗸 | ? |
| 1. Were control and treatment groups comparable at entry? | 🗸 | 🗸 | 🗸 | 🗸 | 🗸 | 🗸 | 🗸 | 🗸 | 🗸 | 🗸 |
| 1. Were groups treated identically other than for the named interventions? | 🗸 | 🗸 | 🗸 | 🗸 | 🗸 | 🗸 | 🗸 | 🗸 | 🗸 | 🗸 |
| 1. Were outcomes measured in the same way for all groups? | 🗸 | 🗸 | 🗸 | 🗸 | 🗸 | 🗸 | 🗸 | 🗸 | 🗸 | 🗸 |
| 1. Were outcomes measured in a reliable way? | 🗸 | 🗸 | 🗸 | 🗸 | 🗸 | 🗸 | 🗸 | 🗸 | 🗸 | 🗸 |
| 1. Was appropriate statistical analysis used? | 🗸 | 🗸 | 🗸 | 🗸 | 🗸 | 🗸 | 🗸 | 🗸 | 🗸 | 🗸 |
| Overall appraisal(I = include, E: exclude and SI: seek further information | I | I | I | I | I | I | I | I | I | I |
| NA = not applicable to community based randomized trials \| ? = unclear | | | | | | | | | | |

##

## JBI critical appraisal checklist for non-randomized experimental studies

| Criteria | Included papers | |
| --- | --- | --- |
|  | Arnold , 2009 | Langford, 2011 |
| 1. Is it clear in the study what is the ‘cause’ and what is the ‘effect’ (i.e. there is no confusion about which variable comes first)? | 🗸 | 🗸 |
| 1. Were the participants included in any comparisons similar? | 🗸 | 🗸 |
| 1. Were the participants included in any comparisons receiving similar treatment/care, other than the exposure or intervention of interest? | SI | SI |
| 1. Was there a control group? | 🗸 | 🗸 |
| 1. Were there multiple measurements of the outcome both pre and post the intervention/exposure? | 🞨 | 🗸 |
| 1. Was follow up complete and if not, were differences between groups in terms of their follow up adequately described and analyzed? | 🗸 | 🗸 |
| 1. Were the outcomes of participants included in any comparisons measured in the same way? | 🗸 | 🗸 |
| 1. Were outcomes measured in a reliable way? | 🗸 | 🗸 |
| 1. Was appropriate statistical analysis used? | 🗸 | 🗸 |
| Overall appraisal (I =Include, E = Exclude, SI = Seek further information) | I | I |
