# Supplementary material for: Effects of single and combined water, sanitation and hygiene (WASH) interventions on nutritional status of children: a systematic review and meta-analysis
Source: Ital J Pediatr. 2019 Jul 4;45:77. doi: 10.1186/s13052-019-0666-2 (PMC6610930; doi:10.1186/s13052-019-0666-2)
Supplement: Supplementary file 2 — Data extraction format. (DOCX 35 kb) [file 13052_2019_666_MOESM2_ESM.docx]

## Data extraction format

| Reviewer Date  Author Year  Journal Record Number | | | | | | |
| --- | --- | --- | --- | --- | --- | --- |
| **Study Method**  RCT □ Quasi-RCT □ Longitudinal □  Retrospective □ Observational □ Other □ | | | | | | |
| **Participants**  Setting  -------------------------------------------------------------------------------------------------------------------------------  Population  ------------------------------------------------------------------------------------------------------------------------------  **Sample size**  Control group _____________________ Experimental group _______________________  **Interventions**  Intervention for the experimental group  -------------------------------------------------------------------------------------------------------------------------------  Intervention for the control group  **------------------------------------------------------------------------------------------------------------------------------**  **Authors conclusion**  ------------------------------------------------------------------------------------------------------------------------------  **Reviewers conclusion**  ------------------------------------------------------------------------------------------------------------------------------ | | | | | | |
| **Study results** | | | | | | |
| Study | Experimental group | | | Control group | | |
|  | Mean | SD | Total | Mean | SD | total |
|  |  |  |  |  |  |  |
|  |  |  |  |  |  |  |
