# Supplementary material for: Effects of single and combined water, sanitation and hygiene (WASH) interventions on nutritional status of children: a systematic review and meta-analysis
Source: Ital J Pediatr. 2019 Jul 4;45:77. doi: 10.1186/s13052-019-0666-2 (PMC6610930; doi:10.1186/s13052-019-0666-2)
Supplement: Supplementary file 3 — Criteria for judging risk of bias in the ‘Risk of bias’ assessment tool. (DOCX 59 kb) [file 13052_2019_666_MOESM3_ESM.docx]

## Criteria for judging risk of bias in the ‘Risk of bias’ assessment tool

| Criteria | **Yes (low risk of bias)** | **No (high risk of bias)** | **Unclear** (uncertain risk of bias) |
| --- | --- | --- | --- |
| **Sequence generation** | **Yes** | **No** | **Unclear** |
| Was the allocation sequence adequately generated? [Short form: *Adequate sequence*  *generation*?] | The investigators describe a random component in the sequence generation process such as:  🡪 Referring to a random number table;  🡪Using a computer random number generator;  🡪Coin tossing;  🡪Shuffling cards or envelopes;  🡪Throwing dice;  🡪Drawing of lots;  🡪Minimization*.  *Minimization may be implemented without a random element, and this is considered to be equivalent to being random. | The investigators describe a non-random component in the sequence generation process. Usually, the description would involve some systematic, non-random approach, for example:  🡪Sequence generated by odd or even date of birth;  🡪Sequence generated by some rule based on date (or day) of admission;  🡪Sequence generated by some rule based on hospital or clinic record number.  Other non-random approaches happen much less frequently than the systematic approaches mentioned above and tend to be obvious.  They usually involve judgement or some method of non-random categorization of participants, for example:  🡪Allocation by judgement of the clinician;  🡪Allocation by preference of the participant;  🡪Allocation based on the results of a laboratory test or a series of tests;  🡪Allocation by availability of the intervention | Insufficient information about the sequence generation process  To permit judgement of ‘Yes’ or ‘No’. |
| **Allocation concealment** | **Yes** | **No** | **Unclear** |
| Was allocation adequately concealed? [Short form: *Allocation concealment*?] | Participants and investigators enrolling participants could not foresee assignment because one of the following, or an equivalent method, was used to conceal allocation:  🡪Central allocation (including telephone, web-based and pharmacy-controlled randomization);  🡪Sequentially numbered drug containers of identical appearance;  🡪Sequentially numbered, opaque, sealed envelopes | Participants or investigators enrolling participants could possibly foresee assignments and thus introduce selection bias, such as allocation based on:  Using an open random allocation schedule (e.g. a list of random numbers); assignment envelopes were used without appropriate safeguards (e.g. if envelopes were unsealed or non-opaque or not sequentially numbered);  Alternation or rotation;  Date of birth;  Case record number;  Any other explicitly unconcealed procedure. | Insufficient information to permit judgement of ‘Yes’ or ‘No’.  This is usually the case if the method of concealment is not described  or not described in sufficient detail to allow a definite  judgement – for example if the use of assignment envelopes is  described, but it remains unclear whether envelopes were sequentially  numbered, opaque and sealed |
| **Blinding of participants, personnel and outcome assessors** | **Yes** | **No** | **Unclear** |
| Was knowledge of the allocated interventions adequately prevented during the study?  [Short form: *Blinding*?] | Any one of the following:  🡪No blinding, but the review authors judge that the outcome and the outcome measurement are not likely to be influenced by lack of blinding;  🡪Blinding of participants and key study personnel ensured, and unlikely that the blinding could have been broken;  🡪Either participants or some key study personnel were not blinded, but outcome assessment was blinded and the non-blinding of others unlikely to introduce bias. | Any one of the following:  🡪No blinding or incomplete blinding, and the outcome or outcome measurement is likely to be influenced by lack of blinding;  🡪 Blinding of key study participants and personnel  attempted, but likely that the blinding could have been broken;  🡪Either participants or some key study personnel were not blinded, and the non-blinding of others likely to introduce bias. | Any one of the following:  🡪Insufficient information to permit judgement of ‘Yes’ or ‘No’;  🡪The study did not address this outcome |
| **Incomplete outcome data** | **Yes** | **No** | **Unclear** |
| Were incomplete outcome data adequately addressed? [Short form: *Incomplete outcome*  *data addressed*?] | Any one of the following:  🡪No missing outcome data;  🡪Reasons for missing outcome data unlikely to be related to true outcome (for survival data, censoring unlikely to be introducing bias);  🡪Missing outcome data balanced in numbers across intervention groups, with similar reasons for missing  data across groups;  🡪For dichotomous outcome data, the proportion of missing outcomes compared with observed event risk not enough to have a clinically relevant impact on the intervention effect estimate;  🡪For continuous outcome data, plausible effect size (difference in means or standardized difference in means) among missing outcomes not enough to have a clinically relevant impact on observed effect size;  🡪Missing data have been imputed using appropriate methods. | Any one of the following:  🡪Reason for missing outcome data likely to be related to true outcome, with either imbalance in numbers or reasons for missing data across intervention groups;  🡪For dichotomous outcome data, the proportion of  missing outcomes compared with observed event risk enough to induce clinically relevant bias in intervention effect estimate;  🡪For continuous outcome data, plausible effect size  (difference in means or standardized difference in  means) among missing outcomes enough to induce  clinically relevant bias in observed effect size;  🡪‘As-treated’ analysis done with substantial departure of the intervention received from that assigned at randomization;  🡪Potentially inappropriate application of simple imputation. | Any one of the following:  Insufficient reporting of attrition/exclusions to permit  judgement of ‘Yes’ or ‘No’ (e.g. number randomized  not stated, no reasons for missing data provided);  The study did not address this outcome |
| **Selective outcome reporting** | **Yes** | **No** | **Unclear** |
| Are reports of the study free of suggestion of selective outcome reporting? [Short form:  *Free of selective reporting*?] | Any of the following:  🡪The study protocol is available and all of the study’s pre-specified (primary and secondary) outcomes that  are of interest in the review have been reported in the pre-specified way;  🡪The study protocol is not available but it is clear that the published reports include all expected outcomes,  including those that were pre-specified (convincing text of this nature may be uncommon). | Any one of the following:  🡪Not all of the study’s pre-specified primary outcomes  have been reported;  🡪One or more primary outcomes is reported using  measurements, analysis methods or subsets of the data (e.g. subscales) that were not pre-specified;  🡪One or more reported primary outcomes were not  pre-specified (unless clear justification for their reporting is provided, such as an unexpected adverse effect);  🡪One or more outcomes of interest in the review are  reported incompletely so that they cannot be entered  in a meta-analysis;  🡪The study report fails to include results for a key outcome that would be expected to have been reported for such a study. | Insufficient information to permit judgement of ‘Yes’ or ‘No’. It  is likely that the majority of studies will fall into this category. |
| **Other potential threats to validity** | **Yes** | **No** | **Unclear** |
| Was the study apparently free of other problems that could put it at a risk of bias?  [Short form: *Free of other bias*?] | The study appears to be free of other sources of bias. | There is at least one important risk of bias. For example, the study:  Had a potential source of bias related to the specific  study design used; or Stopped early due to some data-dependent process (including a formal-stopping rule); or Had extreme baseline imbalance; or Has been claimed to have been fraudulent; or Had some other problem. | There may be a risk of bias, but there is either:  🡪Insufficient information to assess whether an important  risk of bias exists; or  🡪Insufficient rationale or evidence that an identified  problem will introduce bias. |
| Summary assessments of the risk of bias for each important outcome (across domains) within and across studies | | | |
| Risk of bias | Interpretation | Within a study | Across studies |
| Low risk of bias | Plausible bias unlikely to seriously  alter the results | Low risk of bias for all key domains. | Most information is from studies at low risk of bias |
| Unclear risk of bias | Plausible bias that raises some doubt  about the results | Unclear risk of bias for one or more key domains | Most information is from studies at low or unclear risk of bias. |
| High risk of bias | Plausible bias that seriously weakens  confidence in the results | High risk of bias for one or more key domains | The proportion of information from  studies at high risk of bias is sufficient to affect the interpretation of results |
